# The complete chloroplast genome of *Securigera varia* (L.) Lassen

**DOI:** 10.1080/23802359.2021.1886018

**Published:** 2021-03-15

**Authors:** Yi Zhao, Qianqian Xi, Xiangyu Wei, Jiayi Fu, Qianqian Wang, Hongyan He, Chen Ling, Tianliang Chang, Yuwei Zhao

**Affiliations:** a Provincial Key Laboratory of Biotechnology of Shaanxi Province, Northwest University, Xi’an, China; b College of Life Sciences, Northwest University, Xi’an, China; c Key Laboratory of Resource Biology and Biotechnology in Western China (Ministry of Education), Northwest University, Xi’an, China

**Keywords:** *Securigera varia*, chloroplast genome, phylogenetic analysis

## Abstract

*Securigera varia* is an important leguminous forage grass species that is mainly distributed in arid and semi-arid land with water scarcity, and has outstanding drought resistance. In this study, Illumina sequencing was used to obtain the complete sequence of the *S. varia* chloroplast genome. The complete genome was 154,257 bp in length with 35.9% GC content. It was a circular genome containing a large single-copy region (LSC, 84,762 bp), a small single-copy region (SSC, 18,059 bp), and a pair of inverted repeat regions (IRs, 51,436 bp). A total of 128 genes were encoded, including 83 protein-coding genes, 37 tRNAs, and 8 rRNAs. Phylogenetic analysis revealed that *S. varia* was closely related to *Robinia pseudoacacia*. The sequence data of *S. varia* chloroplast genome could provide useful genetic information for the studies on phylogenetic and evolutionary of Leguminosae.

*Securigera varia* is classified as a perennial grass species in the Leguminosae family. This weed has been domesticated as forage or as a landscape plant for its strong stress resistance and delicate pastel-colored flowers (Qian et al. 2015; Zheng et al. 2016). Therefore, *S. varia* has been introduced and cultivated in meadows of many countries, such as the United States, China, Russia, Australia, and Canada (Symstad 2004). The natural habitat of this plant is mainly arid and semi-arid land deficient of irrigation water and with a complex geological environment and climatic conditions (Tabari 2012). Compared to its relative leguminous forage species, such as *Medicago sativa* L., *S. varia* has outstanding drought-stress resistance to prevent damage to vegetative growth caused by water deficiency and rapid recovery of metabolic activities during the rehydration process after drought stress (Debaeke and Aboudrare 2004). However, there are few reports about the genetic evolution analysis of *S. varia*. In this study, we reported the complete chloroplast genome of *S. varia*, which will provide useful genetic information for the studies on phylogenetic and evolutionary of Leguminosae.

Fresh leaves of *S. varia* were harvested from 3 different plantlets at the Botanical Garden of Northwest University (34°25′N, 108°93′E). A specimen was deposited at the Northwest University herbarium (Yuwei Zhao, zhaoyw@nwu.edu.cn) under the voucher number 2020021, and DNA samples were stored at −80 °C at the Provincial Key Laboratory of Biotechnology of Shaanxi Province, Xi’an, China. Total genomic DNA was extracted from fresh leaves using the modified CTAB procedure (Doyle 1987), and the chloroplast genome of *S. varia* was sequenced by Illumina NovaSeq PE150 instrument with the 150 paired-ends reading strategy at Beijing Biomarker Technologies Co., Ltd., the complete chloroplast genome was assembled using GetOrganelle program (Jin et al. 2018) with reference to *Glycine max* (NC007942), annotated using Geneious (version 8.0.2), and then was submitted to GenBank with an accession number: MW125582.

The *S. varia* circular chloroplast genome was 154,257 bp and contained a large single-copy region (LSC) with a length of about 84,762 bp and a small single-copy region (SSC) with a length of about 18,059 bp. These two regions were separated by a pair of reverse repeat regions (IRs, 51,436 bp). There were 128 genes in the chloroplast genome containing 83 protein-coding genes, 37 tRNA genes, and 8 rRNA genes. The overall GC content of the entire chloroplast genome was 35.9%.

To determine the phylogenetic position of *S. varia* within the family Leguminosae, the phylogenetic analysis was performed using MEGA 7.0 (Sudhir et al. 2016) with 1000 bootstrap replicates, while the complete genome sequences of 13 other species in Leguminosae, and two outgroup species in Cruciferae were used as references (Peng et al. 2019). The result demonstrated that *S. varia* was closely related to *Robinia pseudoacacia* (Figure 1).

**Figure 1.**
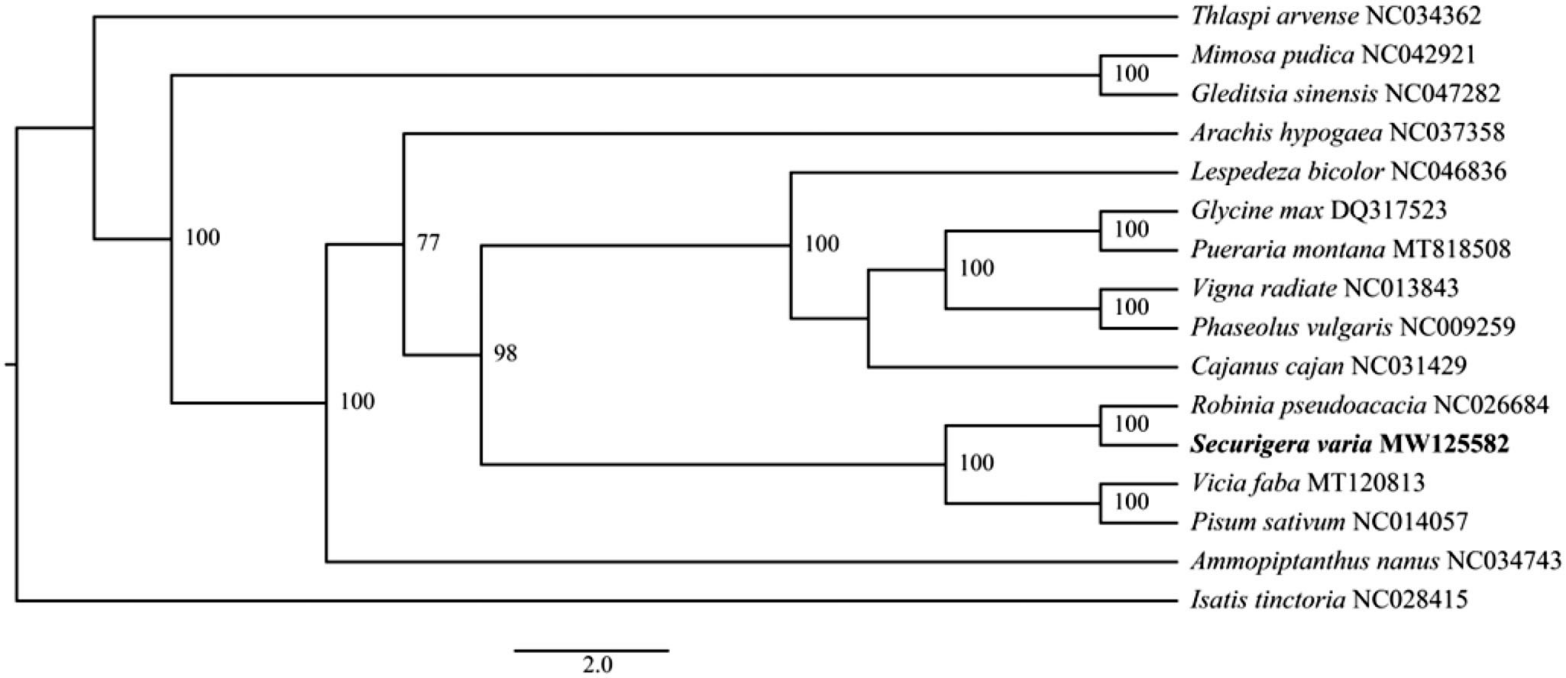
Neighbor-joining phylogenetic tree based on the complete chloroplast genome sequences from fifteen species.

## Data Availability

The genome sequence data that support the findings of this study are openly available in GenBank of NCBI at (https://www.ncbi.nlm.nih.gov/) under the accession number: MW125582. The associated BioProject, SRA, and Bio-Sample numbers are PRJNA689364, SRR13447507 and SAMN17207385, respectively.
